# Therapeutic Outcomes and Toxicity Mitigation of Proton Beam Therapy in Pediatric Neuro-Oncology: A Systematic Review

**DOI:** 10.7759/cureus.93290

**Published:** 2025-09-26

**Authors:** Krina D Patel, Nadiya A Persaud, Sapna Rama

**Affiliations:** 1 Research, Orlando College of Osteopathic Medicine, Winter Garden, USA; 2 Primary Care, Orlando College of Osteopathic Medicine, Winter Garden, USA

**Keywords:** neurocognitive outcomes, pediatric brain tumors, proton beam therapy, proton therapy, radiation toxicity, treatment efficacy

## Abstract

Pediatric brain tumors present unique therapeutic challenges due to their close proximity to critical structures and the vulnerability of the developing brain. Proton beam therapy (PBT) is a modern radiation technique designed to deliver targeted doses to tumors while minimizing exposure to surrounding healthy tissues, offering potential advantages over traditional radiation methods. This systematic review evaluates the efficacy of PBT in treating pediatric brain cancers. A comprehensive search of PubMed was conducted in accordance with the Preferred Reporting Items for Systematic Reviews and Meta-Analyses (PRISMA) guidelines to identify relevant studies. Inclusion criteria required original research published within the past 10 years involving patients under 21 years of age with primary brain tumors of any type treated with PBT. Ten studies met the eligibility criteria and were included in the final analysis. PBT demonstrated equivalent tumor control and survival outcomes compared to conventional therapies while offering significantly reduced risks of radiation-induced toxicities. However, most included studies were retrospective in nature, with limited sample sizes and follow-up duration. PBT appears to be a safe and effective treatment modality for pediatric brain tumors, offering meaningful advantages in toxicity reduction. However, further prospective, randomized studies are needed to strengthen the evidence base, assess long-term outcomes, and evaluate cost-effectiveness to guide future clinical practices.

## Introduction and background

Treatment modalities in pediatric oncology have made much progress over the years, with new methods being tested in order to achieve increased tumor control while minimizing overall patient harm from future complications [1]. Pediatric cancers, specifically brain-related, represent a vulnerable population with complex clinical challenges that subject young patients to harmful effects from traditional radiation therapy in very critical structures of developing children [2]. 

Various photon-based radiotherapy techniques, including intensity-modulated radiotherapy (IMRT), three-dimensional conformal radiotherapy (3D-CRT), and volumetric modulated arc therapy (VMAT), have been the traditional standard of treatment for pediatric brain tumors. Although these techniques allow dose shaping around tumor volumes, the inherent limitation involves significant radiation exposure to adjacent healthy tissues [3]. This exposure has been associated with endocrine dysfunction, neurocognitive decline, growth disturbances, and secondary malignancies in long-term survivors [4]. There are more targeted options, such as brachytherapy and stereotactic radiosurgery (SRS), that have demonstrated excellence in select cases with small or localized lesions [5]. However, they have been limited in broader pediatric application due to risks of invasiveness and radionecrosis. While there are a variety of modalities that offer tumor control benefits, the toxicity profiles of the treatment methods emphasize the critical need for treatment methods that minimize harm in developing children. 

**Figure 1:**
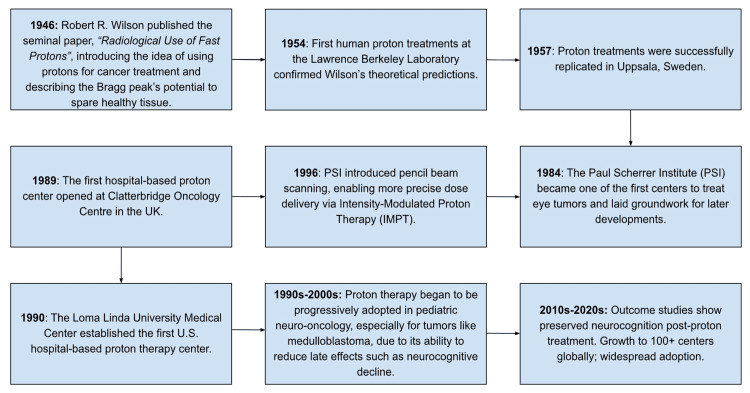
Historical Development of Proton Beam Therapy and its Adoption in Pediatric Neuro-Oncology Infographic designed by Nadiya A. Persaud [8-13]; Software: Canva Pro (Canva Inc., Perth, Australia)

Proton therapy is particularly targeted for tumors in vital complex structures, such as gliomas near the brain, because of the greater direct dose target towards malignant tissues while sparing normal tissue with a lower dose distribution [6]. With the introduction of proton beam therapy (PBT) as a promising alternative to traditional radiation therapies, there has been considerable discussion about whether this new treatment modality inherently produces better long-term outcomes for patients [7]. The historical development of PBT and its adoption in pediatric neuro-oncology is showcased in Figure 1 [8-13].

When a beam of protons enters a medium, the protons gradually lose energy as the depth of the penetration increases. As the protons reach a stopping point, the amount of energy expended occurs more rapidly, creating the Bragg curve [14]. The technique of PBT is theoretically a superior choice for treating cancers in critical locations because protons deposit most of their energy at a specific depth, minimizing impact to normal surrounding tissue structures. Compared to conventional radiation methods such as using photons, PBT provides a more focused dose directly to malignant tissues with fewer doses striking normal surrounding tissues [15]. This feature, in turn, promotes better outcomes for patients with potentially less acute and chronic harmful effects, which is a critical consideration for pediatric populations, given the costly nature of PBT [15].

While theoretically the benefits of PBT may seem advantageous compared to traditional radiation therapies, the practical efficacy and safety of PBT on pediatric patients need to be thoroughly assessed before deeming PBT as the greater treatment modality, given the costly price of the therapy [16]. The therapeutic value depends on a variety of factors, including treatment planning, type of malignancy, dosimetric edge, and availability. Additionally, it is important to understand the statistical outcomes of tumor control, survival rates, and quality of life measures, especially neurocognitive and functional preservation in pediatric brain cancer patients, when deciding the most appropriate therapy route [16].

This systematic review aims to thoroughly assess the current literature published on the efficacy and outcomes of proton therapy in pediatric populations with various brain cancers. We seek to provide an evaluation of how PBT affects tumor control, toxicity profiles, survival rates, and long-term outcomes for pediatric brain cancer populations. With the assessment, this review intends to highlight gaps in current evidence for future research and provide informed clinical practice to support evidence-based implementation of PBT for standard pediatric brain cancer treatment.

Research question and study aims

This study incorporates a review of previous research to determine whether PBT is an effective treatment for pediatric brain cancer in comparison to conventional radiation modalities such as IMRT and 3D-CRT. 

Methods

Search Strategy

This study was conducted in strict accordance with the Preferred Reporting Items for Systematic Reviews and Meta-Analyses (PRISMA) guidelines. A comprehensive literature search was conducted in PubMed using predefined eligibility criteria to filter study titles. Boolean operators were applied to refine the search strategy. The search string used was (“proton therapy” OR “proton beam therapy” OR “proton radiotherapy”) AND (“pediatric brain neoplasms” OR “pediatric brain cancer” OR “pediatric brain tumor” OR “pediatric CNS tumor” OR “pediatric central nervous system neoplasms”). Duplicates and studies unrelated to proton therapy or pediatric brain neoplasms were excluded from the review. 

Inclusion Criteria

The study population included male and female patients <21 years of age diagnosed with primary brain neoplasms. Eligible study designs included randomized controlled trials (RCTs), cohort studies, observational studies, and clinical trials. The primary intervention of interest was proton therapy in comparison to standard photon-based therapies, including 3D-CRT and IMRT, as well as other conventional modalities. Outcomes were evaluated based on efficacy measures as reported by the included studies. Only articles published in English and within the past 10 years (2015-2025) were considered for inclusion. 

Exclusion Criteria

Studies were excluded if they did not report on proton therapy as an intervention or did not involve pediatric/adolescent populations. Ineligible study designs included non-randomized controlled trials, case reports, systematic reviews, narrative reviews, scoping reviews, and studies involving animal subjects. Additionally, studies published in languages other than English or published prior to 2015 were excluded from this review.

Reporting Bias

Covidence software was used to conduct this review. Two independent reviewers assessed titles, abstracts, and full-text articles based on the predefined eligibility criteria. Any disagreements were adjudicated by a senior author. Studies were excluded for reasons such as irrelevant topic, poor study quality, duplicate studies, incorrect intervention (not specific to proton therapy), inappropriate study design (i.e., case reports, non-original studies), or publication in a non-English language. All exclusions were documented within Covidence (Veritas Health Innovation Ltd., Melbourne, Australia) and reflected in the PRISMA flow diagram.

To minimize the risk of reporting bias, potential sources of bias such as publication bias, selective outcome reporting bias, language bias due to the exclusion of non-English studies, and time-lag bias were considered throughout the review process. These risks were acknowledged, and efforts were made to mitigate their impact.

## Review

Results

**Figure 2:**
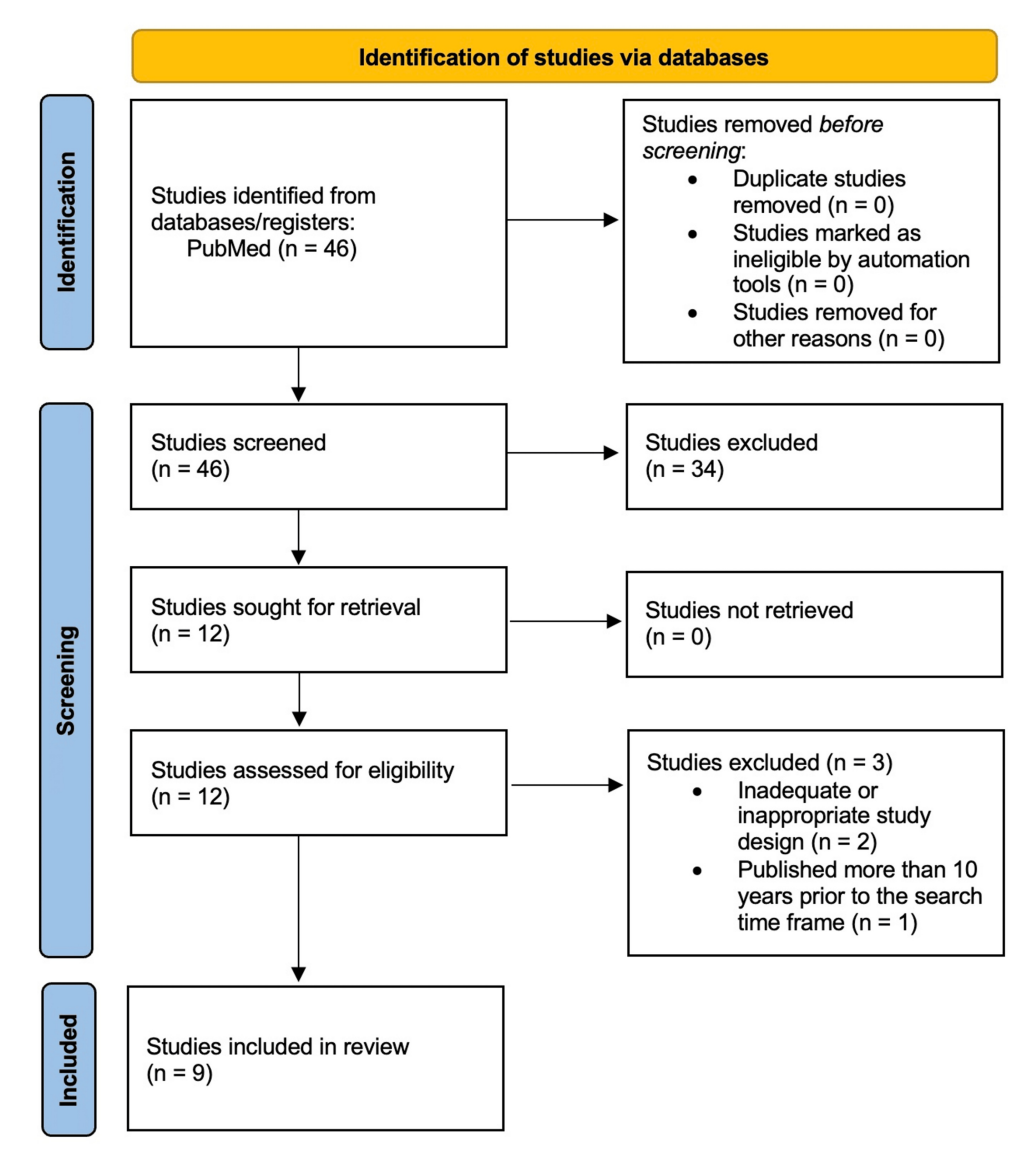
PRISMA Flow Diagram Illustrating the Study Selection Process for Inclusion of Articles Evaluating Proton Therapy in Pediatric Brain Tumors PRISMA 2020 flow diagram created using the PRISMA online tool, summarizing study selection and exclusion based on predefined criteria. PRISMA: Preferred Reporting Items for Systematic Reviews and Meta-Analyses

A total of 46 studies were initially identified through a PubMed search based on predefined eligibility criteria. After conducting the title and abstract screening, 34 studies were excluded for irrelevance. Of the 12 studies that progressed to full-text review, two were excluded due to ineligible study designs, and one was excluded for being published more than 10 years before the defined search time frame. Ultimately, nine studies met the inclusion criteria and were included in this systematic review (Figure 2).

The included studies predominantly focused on pediatric and adolescent patients (≤21 years old) diagnosed with various brain neoplasms. Proton therapy was assessed either as the primary treatment or in combination with other interventions, including surgery and chemotherapy. Comparative treatments included conventional photon-based modalities such as IMRT and 3D-CRT.

Overall, the nine included studies encompassed a combined total of 637 pediatric patients (≤21 years old) treated for various brain neoplasms, including medulloblastoma, craniopharyngioma, ependymoma, low-grade glioma, and germinoma. Across these studies, PBT was associated with higher rates of disease control and survival, lower radiation exposure to healthy brain structures, and reduced risks of long-term toxicities such as ototoxicity, neuroendocrine dysfunction, and neurocognitive decline compared to historical or photon-based controls. 

Discussion

Tumor Control Across Pediatric Brain Tumors

Across the 10 studies that met inclusion criteria, proton therapy consistently demonstrated effectiveness in tumor control while significantly reducing radiation-induced toxicity compared to conventional photon-based modalities such as IMRT and 3D-CRT (Table 1). This finding was consistent across tumor types, including medulloblastomas, ependymomas, low-grade gliomas, craniopharyngiomas, and germinomas.

**Table 1:** Summary of Included Studies Evaluating the Efficacy and Toxicity Outcomes of Proton Therapy for Pediatric Brain Tumors

Title	Reference	Population	Intervention	Comparison	Outcome	Research Question	Study Design	Sample Size	Findings
Proton therapy and limited surgery for paediatric and adolescent patients with craniopharyngioma (RT2CR): a single-arm, phase 2 study.	Merchant TE, Hoehn ME, Khan RB, et al. [17]	Pediatric patients (21 years and younger) diagnosed with intracranial ependymoma.	Proton beam radiation therapy (PBRT)	Intensity modulated radiation therapy (IMRT)	Incidence and types of imaging changes (cyst formation, T2/Fluid-attenuated inversion recovery (FLAIR) hyperintensities), amount of time it took to observe imaging changes on follow-up MRIs, and appearance and location of imaging abnormalities within the brain.	Does the combination of limited surgery and PBRT improve progression-free survival, overall survival, and cognitive outcomes in pediatric and adolescent patients with craniopharyngioma compared to historical data from photon therapy?	Retrospective	41 patients, 23 received PBRT, 18 received IMRT	This study demonstrates that limited surgery followed by PBRT offers high rates of tumor control with minimal severe complications in pediatric and adolescent patients with craniopharyngioma. While survival outcomes are comparable to those achieved with photon therapy, PBRT is associated with improved cognitive outcomes, likely due to reduced radiation exposure to healthy brain tissue. Specifically, patients treated with PBRT showed no significant annual decline in IQ or adaptive behavior, whereas those receiving photon therapy experienced mean declines of 1.09 IQ points and 1.48 adaptive behavior points per year.
Imaging Changes in Pediatric Intracranial Ependymoma Patients Treated With Proton Beam Radiation Therapy Compared to Intensity Modulated Radiation Therapy.	Gunther JR, Sato M, Chintagumpala M, et al. [18]	Pediatric patients (21 years and younger) with newly diagnosed craniopharyngioma who had not received radiotherapy or intracystic therapy	Proton therapy using a 0.5 cm clinical target volume margin and a total dose of 54 Gy	Pediatric patients treated with photon radiotherapy	Incidence, timing, and resolution of post-radiation MRI changes; clinical significance of these changes	Does PBRT result in a higher incidence of post-radiation MRI changes compared to IMRT in pediatric patients with intracranial ependymoma, and what is the clinical significance of these changes?	Retrospective	72 pediatric patients; PBRT group: 37 patients, IMRT group: 35 patients	This study indicates a higher incidence of post-radiation MRI changes in pediatric ependymoma patients treated with PBRT compared to IMRT. While many of these changes are self-limiting, some can lead to significant clinical symptoms requiring intervention. These findings highlight the need for careful monitoring of patients undergoing PBRT and consideration of potential risks when choosing the radiation modality.
Increased risk of pseudoprogression among pediatric low-grade glioma patients treated with proton versus photon radiotherapy.	Ludmir EB, Mahajan A, Paulino AC, et al. [19]	83 pediatric patients (≤21 years) with low-grade glioma (LGG)	Proton beam therapy (PBT)	Photon-based IMRT	Incidence of pseudoprogression (PsP) post-radiotherapy	Does PBT increase the risk of pseudoprogression compared to photon-based IMRT in pediatric patients with low-grade glioma?	Retrospective	83 pediatric patients; IMRT group: 32 patients, PBT group: 51 patients	This study highlights a higher incidence of pseudoprogression in pediatric LGG patients treated with PBT compared to IMRT. The findings suggest that clinicians should be aware of the increased likelihood of PsP with PBT to avoid misinterpretation as tumor progression, which could lead to unnecessary interventions.
Long-term toxic effects of proton radiotherapy for paediatric medulloblastoma: a phase 2 single-arm study.	Yock TI, Yeap BY, Ebb DH, et al. [20]	59 pediatric patients aged three to 21 years diagnosed with medulloblastoma	Proton radiotherapy (PRT) following surgical resection and chemotherapy	No direct comparison group	Assessment of long-term toxic effects, including ototoxicity, neuroendocrine dysfunction, and neurocognitive outcomes; evaluation of disease control and overall survival	What are the long-term toxic effects and clinical outcomes associated with PRT in pediatric patients with medulloblastoma?	Non-randomized, open-label, single-center, phase 2 single-arm trial	59 pediatric patients	PRT in pediatric medulloblastoma patients resulted in acceptable long-term toxic effects, with lower rates of severe ototoxicity and endocrine dysfunction compared to historical photon therapy data. The study demonstrated favorable survival outcomes, supporting the efficacy of proton therapy in disease control.
A comparative study of dose distribution of PBT, 3D-CRT and IMRT for pediatric brain tumors.	Takizawa D, Mizumoto M, Yamamoto T, et al. [21]	12 pediatric patients 6 w/ ependymoma (requiring local irradiation) 6 w/ germinoma (requiring whole-ventricle irradiation)	PBT	Three-dimensional conformal radiotherapy (3D-CRT) and IMRT	Comparison of dose distribution to normal brain tissue among the three modalities	Does PBT offer superior sparing of normal brain tissue compared to 3D-CRT and IMRT in pediatric patients undergoing radiotherapy for brain tumors?	Retrospective	12 pediatric patients; Ependymoma group: 6 patients, Germinoma group: 6 patients	PBT offers superior sparing of normal brain tissue compared to both 3D-CRT and IMRT in pediatric brain tumor patients. The lack of significant difference between 3D-CRT and IMRT in terms of normal brain dose suggests that PBT may be the preferred modality when available, especially in pediatric populations, where minimizing radiation exposure to healthy tissue is paramount.
Clinical Outcomes Among Children With Standard-Risk Medulloblastoma Treated With Proton and Photon Radiation Therapy: A Comparison of Disease Control and Overall Survival.	Eaton BR, Esiashvili N, Kim S, et al. [22]	88 children diagnosed with standard-risk medulloblastoma, aged three to 21 years	Postoperative PRT	Postoperative PRT	Postoperative PRT	Does PRT provide equivalent disease control and overall survival compared to PRT in children with standard-risk medulloblastoma?	Retrospective	88 children Proton therapy group: 45 patients Photon therapy group: 43 patients	This study demonstrates that proton therapy offers disease control and overall survival rates comparable to photon therapy in children with standard-risk medulloblastoma. The findings support the use of proton therapy as an effective treatment modality, with the added potential benefit of reduced radiation exposure to surrounding healthy tissues.
Prospective, longitudinal comparison of neurocognitive change in pediatric brain tumor patients treated with proton radiotherapy versus surgery only.	Kahalley LS, Ris MD, Mahajan A, et al. [16]	93 pediatric brain tumor patients aged ≤21 years	PRT, subdivided into: proton craniospinal irradiation (P-CSI) and proton focal radiotherapy (P-Focal)	Surgery only (SO) group	Longitudinal changes in neurocognitive functions, specifically: Full Scale IQ (FSIQ), Verbal Comprehension Index (VCI), Perceptual Reasoning Index (PRI), Working Memory Index (WMI), Processing Speed Index (PSI)	How does PRT, both craniospinal and focal, impact neurocognitive outcomes over time in pediatric brain tumor patients compared to surgery alone?	Prospective, longitudinal cohort study	93 patients, 22 received P-CSI, 31 received P-Focal, 40 underwent surgery only	P-Focal demonstrated neurocognitive outcomes comparable to surgery alone, suggesting its safety in preserving cognitive functions. P-CSI was associated with declines in specific cognitive domains, indicating a need for careful consideration when selecting treatment modalities.
Outcomes following proton therapy for pediatric ependymoma.	Indelicato DJ, Bradley JA, Rotondo RL, et al. [23]	179 pediatric patients (≤21 years old) with nonmetastatic WHO grade II or III intracranial ependymoma	Postoperative proton therapy	No direct comparison group; outcomes contextualized against historical photon therapy data	Assessment of disease control (local control, progression-free survival, overall survival) and treatment-related toxicity	Does postoperative proton therapy provide effective disease control with acceptable toxicity profiles in pediatric patients with nonmetastatic intracranial ependymoma?	Retrospective	179 patients, 74 diagnosed with grade II tumors, 105 diagnosed with grade III tumors	This study supports the efficacy of proton therapy in managing pediatric intracranial ependymoma, with three-year local control of 83% and overall survival of 95%, rates comparable to photon therapy. Importantly, the toxicity profile was favorable, with clinically significant brainstem injury observed in only 2% of patients.
Temporal lobe sparing radiotherapy with photons or protons for cognitive function preservation in paediatric craniopharyngioma.	Toussaint L, Indelicato DJ, Muren LP, et al. [24]	Pediatric craniopharyngioma patients previously treated with double-scattering proton therapy (DSPT)	Radiotherapy techniques with temporal lobe sparing included volumetric modulated arc therapy (VMAT) using photons, and DSPT and PBS using protons, each delivering treatment while aiming to minimize radiation exposure to surrounding healthy tissue.	Comparison among three radiotherapy modalities: VMAT, DSPT, and pencil beam scanning (PBS) proton therapy	Assessment of radiation dose exposure to critical brain structures, particularly the temporal lobes and hippocampi, with implications for cognitive function preservation	Does the use of temporal lobe sparing radiotherapy techniques, specifically proton therapy modalities like DSPT and PBS, reduce radiation exposure to critical brain structures compared to photon-based VMAT in pediatric patients with craniopharyngioma, thus potentially preserving cognitive function?	Retrospective	10 patients diagnosed with high-grade glioma	This study provides evidence that proton therapy, especially PBS, offers superior sparing of critical brain structures involved in cognition compared to traditional photon therapy (VMAT). In a cohort of 179 children with intracranial ependymoma (median age 3.5 years; 67% WHO grade III; 66% posterior fossa), proton therapy achieved 3-year local control, progression-free survival, and overall survival rates of 85%, 76%, and 90%, respectively.

Medulloblastoma Outcomes

Proton therapy provided disease control and survival outcomes comparable to photon-based therapies in children with standard-risk medulloblastomas and intracranial ependymomas. Eaton et al. found no significant differences in progression-free survival between the two groups, supporting the clinical efficacy of proton therapy in this population while potentially minimizing radiation exposure to healthy tissues [22]. Similarly, Yock et al. demonstrated favorable long-term outcomes with proton therapy in medulloblastoma, reporting acceptable rates of ototoxicity and neuroendocrine dysfunction, particularly when compared to historical photon therapy data [20].

Ependymoma Outcomes

In the treatment of pediatric ependymomas, multiple studies emphasized the long-term advantages of proton therapy. Indelicato et al. observed effective disease control with postoperative proton therapy in 179 children, noting a favorable toxicity profile and reduced radiation exposure to normal brain tissues [20]. Gunther et al. compared imaging changes in patients receiving proton therapy versus IMRT and found that while proton beam radiation therapy (PBRT) was associated with a higher incidence of MRI changes, these were mostly self-limiting; nevertheless, the findings underscore the importance of ongoing surveillance [18].

Neurocognitive and Functional Preservation

Neurocognitive preservation emerged as a strong benefit of proton therapy. In a longitudinal study, Kahalley et al. found that patients who underwent focal proton therapy (P-Focal) had neurocognitive outcomes comparable to surgery-only controls, suggesting a strong protective effect of proton therapy on brain function. However, craniospinal proton therapy (P-CSI) was associated with cognitive decline in specific domains, highlighting the need for judicious application [16]. Similarly, Toussaint et al. reported superior sparing of the temporal lobes and hippocampi using pencil beam scanning (PBS) proton therapy over photon-based VMAT, reinforcing the role of proton therapy in reducing the risk of radiation-induced cognitive impairment [24].

Endocrine, Ototoxicity, and Craniopharyngioma Outcomes

Reduced negative impact of memory issues, hearing loss, and endocrine dysfunction were linked to proton therapy treatment. Yock et al. documented lower rates of severe ototoxicity and neuroendocrine complications, likely due to the conformal nature of proton dose distribution [25-26]. These findings are especially crucial amongst the pediatric population, where long-term endocrine health is a growing concern. In craniopharyngioma patients, Merchant et al. reported that limited surgery followed by PBRT resulted in improved control rates with minimal complications and better cognitive preservation compared to IMRT [17].

Dosimetric Advantages

Dosimetric studies consistently demonstrate that proton therapy delivers lower integral doses to healthy tissue. Takizawa et al. showed that proton therapy provided significantly reduced exposure to surrounding healthy brain tissues compared to both 3D-CRT and IMRT, supporting its superiority in minimizing off-target effects [21]. This is particularly important for tumors located near critical brain structures.

Limitations of Current Evidence

Despite these promising results, there were several limitations that surfaced. The majority of included studies in this review were observational and retrospective, with relatively small sample sizes. The volume of RCTs was deficient, and only a limited number of prospective trials were found. Furthermore, the literature systematic review’s scope was constrained by its dependence on only literature indexed in PubMed. This dataset may have been further expanded with the inclusion of databases such as Scopus or the Excerpta Medica database (EMBASE). Additionally, long-term follow-up figures were oftentimes insufficient to draw further detailed conclusions on the extent of greater clinical outcomes of proton therapy when compared to traditional cancer treatment methods. There was also a lack of attention on the cost-effectiveness of proton therapy compared to traditional treatment modalities, which should be a principal consideration in terms of broader healthcare implementation. 

In addition to these clinical considerations, accessibility and cost remain major barriers to the widespread use of PBT. Although treatment outcomes have been impressive, PBT remains more expensive than photon IMRT due to the high capital and operational costs of building and maintaining proton facilities [26-27]. However, cost-effectiveness analyses, including studies in pediatric medulloblastoma, suggest that PBT may provide better long-term economic value by reducing late toxicities and improving quality of life [26-27]. Access also remains limited by geographic constraints and infrastructure requirements [28]. Future research should continue to evaluate both clinical outcomes and cost-effectiveness to support equitable access and resource allocation.

Clinical Implications and Future Research

Overall, this review highlights PBT as a promising advancement in pediatric neuro-oncology. Across tumor types, PBT provides disease control comparable to conventional photon-based therapies while significantly reducing long-term toxicities such as neurocognitive decline, endocrine dysfunction, and ototoxicity. These benefits are particularly important in the developing pediatric population, where treatment-related late effects can have lifelong consequences. The dosimetric advantages of PBT, including reduced exposure to adjacent normal tissues, further reinforce its role in treating tumors located near critical brain structures [17].

Further studies, such as prospective trials and registry-based cohorts, are essential to demonstrate the clinical benefit of PBT in pediatric brain tumors. Given the rarity of these cancers and the ethical challenges of randomization, long-term comparative data using cohort designs are particularly valuable. Despite increased uptake, no level-I randomized evidence yet supports PBT’s superiority in this setting [29].

## Conclusions

PBT serves as a promising advancement in pediatric neuro-oncological treatment, offering the potential for effective tumor control with significantly reduced radiation-induced toxicities compared to conventional photon-based modalities. This systematic review demonstrates that PBT provides comparable survival outcomes to traditional therapies while minimizing long-term complications such as neurocognitive decline and endocrine dysfunction. This is especially imperative amongst the developing pediatric population. Dosimetric advantages of PBT support its use in treating tumors located near sensitive brain structures, reinforcing its clinical utility in complex cases. Overall, the included studies provide strong evidence supporting proton therapy as an effective treatment for pediatric brain neoplasms, highlighting improved clinical outcomes, reduced radiation-related toxicity, and better preservation of neurocognitive function compared to conventional photon-based therapies. However, limitations in the current body of evidence, particularly the overrepresentation of retrospective designs, small sample sizes, limited long-term follow-up, and lack of cost-effectiveness analyses, emphasize the need for further prospective, randomized trials. As access to proton therapy expands, robust clinical evidence will be essential to guide its implementation in standard pediatric brain cancer treatment protocols and to ensure equitable, evidence-based care for this vulnerable patient population.
